# Nitric oxide-generating compound GSNO suppresses porcine circovirus type 2 infection in vitro and in vivo

**DOI:** 10.1186/s12917-017-0976-9

**Published:** 2017-02-21

**Authors:** Chuanmin Liu, Libin Wen, Qi Xiao, Kongwang He

**Affiliations:** 1 Institute of Veterinary Medicine, Jiangsu Academy of Agricultural Sciences, 50 Zhong-ling Street, Xuanwu District, Nanjing, 210014 China; 2 Key laboratory of Veterinary Biological Engineering and Technology, Ministry of Agriculture, 50 Zhong-ling Street, Xuanwu District, Nanjing, 210014 China; 3 National Center for Engineering Research of Veterinary Bio-products, 50 Zhong-ling Street, Xuanwu District, Nanjing, 210014 China; 4 Jiangsu Co-innovation Center for Prevention and Control of Important Animal Infectious Diseases and Zoonoses, 12 Wen-Hui East Road, Hanjiang District, Yangzhou, 225009 China

**Keywords:** Nitric oxide, GSNO, PCV2, PK-15 cells, BALB/c mice

## Abstract

**Background:**

Nitric oxide (NO), an important signaling molecule with biological functions, has antimicrobial activity against a variety of pathogens including viruses. To our knowledge, little information is available about the regulatory effect of NO on porcine circovirus type 2 (PCV2) infection. This study was conducted to investigate the antiviral activity of NO generated from S-nitrosoglutathione (GSNO), during PCV2 infection of PK-15 cells and BALB/c mice.

**Results:**

GSNO released considerable NO in the culture medium of PK-15 cells, and NO was scavenged by its scavenger hemoglobin (Hb) in a dose-dependent manner. NO strongly inhibited PCV2 replication in PK-15 cells, and the antiviral effect was reversed by Hb. An in vivo assay indicated that GSNO treatment reduced the progression of PCV2 infection in mice, evident as reductions in the percentages of PCV2-positive sera and tissue samples and in the viral DNA copies in serum samples. GSNO also improved the growth performance and immune organs (spleens and thymuses) of the PCV2-infected mice to some degree.

**Conclusions:**

Our data demonstrate that the NO-generating compound GSNO suppresses PCV2 infection in PK-15 cells and BALB/c mice, indicating that NO and its donor, GSNO, have potential value as antiviral drugs against PCV2 infection.

## Background

Porcine circovirus type 2 (PCV2), which belongs to the family *Circoviridae*, is a small, non-enveloped virus with a circular, single-stranded DNA genome [1]. PCV2 is the primary causative agent of porcine circovirus-associated disease (PCVAD) [2], a globally emerging disease, currently causing great economic losses in the global swine industry today [3]. The most significant pathological conditions considered to be PCVADs are post-weaning multisystemic wasting syndrome, porcine dermatitis and nephropathy syndrome, and PCV2-reproductive disease [4, 5]. PCV2-infected piglets are readily contract concomitant infections, including porcine respiratory and reproductive syndrome virus, porcine parvovirus and *Haemophilus parasuis*, suggesting that PCVAD is actually an immunosuppressive disease [6]. Besides piglets, mice and calves are also found to be able to infect this virus [7, 8]. The control of PCVD is based on management strategies, control of coinfections, and vaccination [9]. Vaccination is traditionally considered the most effective method for preventing viral diseases, but the period of protection afforded by a vaccine is limited and the virus cannot be eradicated by vaccination [10]. Furthermore, no effective vaccines are available for preventing multifactorial diseases such as PCVAD [11]. Therefore, alternative effective measures to control the disease are urgently required.

NO is an important molecule with key roles in a broad range of biological processes including neurotransmission, vasodilatation and immune responses [12]. NO is generated by mammalian cells from the guanidino nitrogen of l-arginine in a reaction catalyzed by a family of NO synthase enzymes [13]. NO can also be released from exogenous donors, such as sodium nitroprusside (SNP), S-nitroso-acetylpenicillamine (SNAP) and GSNO [14]. Previous studies have presented considerable evidence that NO can prevent viral infections [15–20]. However, the antiviral effects of NO against PCV2 infection are so far poorly studied.

In this study, the NO-generating compound GSNO was used to analyze the kinetics of NO production in the culture supernatant of PK-15 cells. The antiviral activity mediated by GSNO during PCV2 infection was also investigated in PK-15 cells and BALB/c mice.

## Methods

### Cells and virus

PCV-free PK-15 cells, purchased from the China Institute of Veterinary Drug Control (Beijing, China), were grown at 37 °C in an atmosphere of 5% CO_2_ in Dulbecco’s modified Eagle’s medium (DMEM; Sigma, USA) supplemented with 10% fetal bovine serum (GIBCO, USA) and 1% penicillin-streptomycin antibiotics (Sangon, China). The PCV2-Haian strain (GenBank accession number: FJ712216.1) is maintained by Institute of Veterinary Medicine, Jiangsu Academy Agricultural Sciences, Jiangsu, China. PCV2 was propagated in PK-15 cells, harvested after incubation for 72 h, and stored at −70 °C until use.

### Experimental design

#### In vitro

The safe concentrations of the drugs were determined in PK-15 cells with a 3-(4,5-dimethylthiazol-2-yl)-2,5-diphenyltetrazolium bromide (MTT) assay, and the kinetics of NO production in the culture supernatant were assessed from 0 to 96 h after drug treatment. To investigate the antiviral activity of the NO-generating compound GSNO during PCV2 infection in vitro, 80% confluent PK-15 cells in 24-well cell culture plates were pretreated with GSNO or GSNO plus hemoglobin (Hb) for 6 h, and then infected with PCV2 (multiplicity of infection (MOI) of 1) in the presence of the drugs for an additional 72 h. Untreated cells, cultured in medium alone, were used as the mock control, and cells infected with PCV2 alone (MOI = 1) were used as the PCV2-infected control. The antiviral activity of GSNO was determined from the appearance of PCV2-infected cells, viral titers, and PCV2 DNA copy numbers. The NO levels in the various groups were also determined.

#### In vivo

Seventy-eight 4-week-old specific-pathogen-free BALB/c mice were purchased from the Comparative Medicine Centre, Yangzhou University, Jiangsu, China. The mice were maintained in isolation rooms, in which the temperature was maintained at 25 °C under a 12-h light cycle. The experimental animals were allowed to acclimatize for 7 days. The protocols for the care and use of animals in this study were approved by the Committee on the Ethics of Animal Experiments of Jiangsu Academy of Agricultural Sciences. The mice were randomly divided into three groups, each containing 26 animals. The mice in group I were injected intraperitoneally with 0.2 ml of phosphate-buffered saline (PBS) and intranasally with 0.02 ml of PBS, and used as negative control(NC). Mice in group II were inoculated intraperitoneally with 0.2 ml of PCV2 (10^5^ TCID_50_/ml) and intranasally with 0.02 ml of PCV2 (10^5^ TCID_50_/ml), and used as the PCV2-infected control. The mice in group III were inoculated intraperitoneally with 0.2 ml of PCV2 (10^5^ TCID_50_/ml) and intranasally with 0.02 ml of PCV2 (10^5^ TCID_50_/ml), and injected intraperitoneally with 0.1 ml of GSNO (10 mM) once daily from 0 to 6 days postinoculation (DPI). To exclude the stress induced by the injection, the mice in groups I and II were also injected intraperitoneally with 0.1 ml of PBS once daily at 0–6 DPI. At -1, 12, 17, 22, 27, and 32 DPI, all the mice were weighed, and necropsies were performed on four mice from each group. At 6 DPI, blood samples were withdrawn from the orbital venous plexus of each mouse to isolate serum for the detection of NO. At the time of necropsy, serum samples and tissue samples were collected and stored at −70 °C for PCV2 polymerase chain reaction (PCR) or real-time PCR. The spleen and thymus from each killed mouse were weighed to calculate the organ indexes. The mice were monitored daily and evaluated for clinical signs throughout the trial.

### Cytotoxicity assay

In this study, we used GSNO (Sigma, USA) as the exogenous NO donor; Hb (Sigma, USA), as the NO scavenger to scavenge the NO released from GSNO. The cytotoxicity of the drugs was evaluated with a MTT assay. In brief, PK-15 cells were seeded in 96-well cell culture plates at a density of 5 × 10^4^ cells/well. When the cells in each well reached 80% confluence, they were washed twice with PBS and treated with different concentrations of the drugs as serial two-fold dilutions, with eight wells for each concentration. After incubation for 72 h, the viability of PK-15 cells was evaluated with a colorimetric MTT assay, as reported previously [11]. The absorbance at 570 nm (A_570_) of each well was measured with a microliter enzyme-linked immunosorbent assay reader (Sunrise, TECAN Co., Switzerland).

### Measurement of NO

NO production was measured with a colorimetric assay using the Griess reaction [21]. Briefly, at various time points during cell culture, the supernatants (100 μl/well) were harvested, and incubated with an equal volume of Griess solution (1% sulfanilamide, and 0.1% naphthyl ethylene diamine dihydrochloride in 5% phosphoric acid) (Sigma, USA) for 10 min at room temperature. The absorbance was read at 540 nm, and the concentrations of NO were determined from a least squares linear regression analysis of a standard curve for sodium nitrite.

### Indirect fluorescence assay (IFA)

PCV2-based IFA was performed according to previously described procedures [22]. In brief, cells were fixed in frozen methanol at 4 °C for 10–15 min. The fixed cells were washed with PBS, and incubated with porcine anti-PCV2 antibody (VMRD, USA) at 37 °C for 1 h. The cells were washed with PBS again, incubated with FITC-conjugated goat anti-pig antibody (Abcam, UK) at 37 °C for 45 min, washed again with PBS, and examined under a fluorescence microscope (Olympus, Japan). The cells positive for PCV2 viral antigens were counted in six fields of view.

### Viral titers

Samples collected from the cell culture plates were frozen and thawed three times, then serially diluted 10-fold in DMEM (1:10 to 1:10^6^). PK-15 cells were seeded in 96-well plates, and when the cells in each well reached 40%–50% confluence, they were inoculated with the diluted samples, with eight wells used for each dilution. After incubation for 48 h, the PCV2-positive samples were detected by IFA, as mentioned above, and the viral titers were calculated by Reed-Muench method [23].

### DNA extraction, PCR and TaqMan-based real-time PCR

DNA was extracted by using the Column Viral DNAout Kit (TIANDZ, Beijing, China), according to the manufacturer’s instructions. The total DNA was stored at −70 °C until use.

To evaluate PCV2 viremia or viral loading in the PCV2-infected tissues of mice, a pair of primers was designed based on the published sequences of PCV2 (forward primer 5′-TTACCGGCGCACTTCGGCAG-3′, reverse primer 5′-ACTCCGTTGTCCCTGAGAT-3′), and PCR was performed with the thermal cycling parameters: 94 °C for 5 min followed by 30 cycles of 94 °C for 1 min, 58 °C for 1 min, and 72 °C for 1.5 min, with a final extension step for at 72 °C for 7 min. The PCR products were subjected to electrophoresis on a 1% agarose gel.

To analyze the viral DNA copy numbers in the cell and serum samples, primers and a TaqMan probe specific for the PCV2 sequence were designed: forward primer 5′-TAAATCTCATCATGTCCACATTCCA-3′, reverse primer 5′-CGTTACCGCTGGAGAAGGAA-3′ and TaqMan probe 5′-[6-FAM]AATGGCATCTTCAACACCCGCCTCT[TAMRA]-3′. Taqman-based real-time PCR was performed on the 7500 Real-Time PCR System (Applied Biosystems, USA) using the thermal cycling parameters: 95 °C for 30 s, 40 cycles of 95 °C for 5 s and 60 °C for 34 s.

### Statistical analysis

The differences among different treatment groups were analyzed and compared by one-way analysis of variance (ANOVA), followed by a least-significant difference test, using the statistical package SPSS ver. 17.0 for Windows. A value of *P* < 0.05 was considered statistically significant.

## Results

### Antiviral activity of GSNO in PK-15 cells

**Fig. 1:**
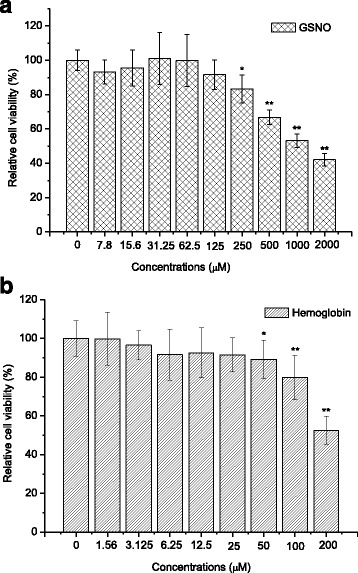
Cytotoxicity of GSNO and Hb on PK-15 cells tested by MTT assay. After incubation with the drugs for 72 h, MTT was added into each well. The cells were cultured for another 4 h, and 200 μl DMSO was added into each well for dissolving formazan, then A_570_ of the samples were determined by a microplate reader. Relative viability was calculated according to the equation: Relative viability (%) = A_570_ of the drug-treated sample / A_570_ of the untreated sample × 100. Data shown were means ± SD from three independent experiments. **a** Cytotoxicity of GSNO on PK-15 cells. **b** Cytotoxicity of Hb on PK-15 cells. ^*^ *P* < 0.05, ^**^ *P* < 0.01 vs untreated control group

As shown in Fig. 1a, the A_570_ value of PK-15 cells treated with 125 μM GSNO did not differ significantly from that of the control cells in the MTT assay (*P* > 0.05). Therefore, 125 μM GSNO was used as a safe concentration for the cells in this study. The safe concentration of Hb for PK-15 cells was determined to be 25 μM (Fig. 1b).

**Fig. 2:**
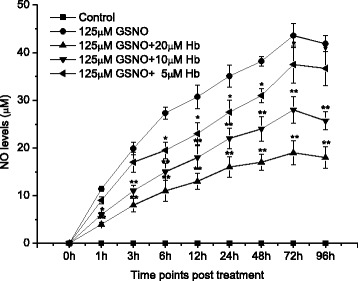
Kinetics of NO production in the culture supernatants of PK-15 cells. When 80% confluent monolayers formed, 125 μM GSNO or 125 μM GSNO plus Hb (5, 10, 20 μM) were added into 96-well cell culture plates, with four wells for each concentration, and untreated cells served as the control. The supernatant from each sample was collected at different time points, and the NO levels were determined by Griess reaction with a microplate reader at 540 nm according to a standard curve made from sodium nitrite. Data were presented as means ± SD from three independent experiments. *P < 0.05, **P < 0.01 vs 125 μM GSNO-treated group

**Fig. 3:**
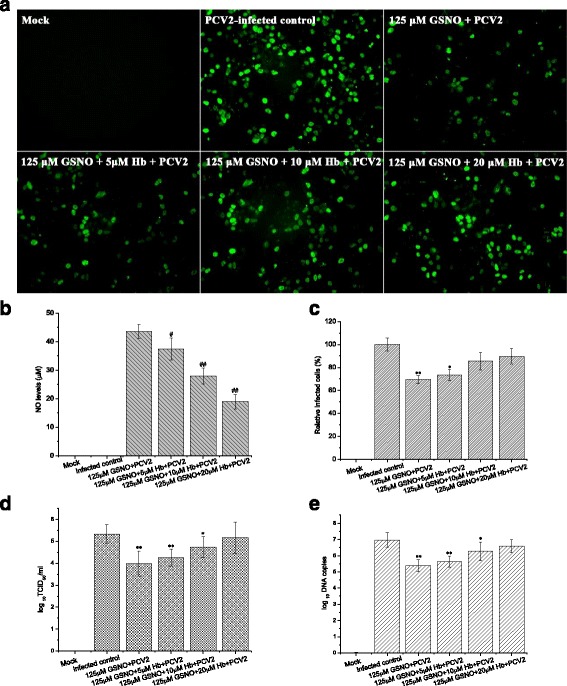
Effects of GSNO on PCV2 replication in PK-15 cells. When monolayers reached about 80% in each well of 24-well cell culture plates, the cells were incubated with 125 μM GSNO or 125 μM GSNO plus Hb (5, 10, 20 μM) for 6 h, with four wells for each treatment, then the cells were infected with PCV2 (1 MOI) in the presence of various drugs for another 72 h. Non-treated cells served as the mock, and the infected cells without drug treatment were considered as the PCV2-infected control. PCV2-positive cells were detected by IFA (**a**), and the appearance of infected cells was judged by FITC staining intensity. The culture supernatant from each well was collected for determination of NO production (**b**), in addition, the cells in each sample were also gathered for assay of the percentage of infected cells (**c**), virus titers (**d**), and viral DNA copies (**e**). Relative infected cells (%) = number of PCV2-positive cells from experimental samples / number of PCV2-positive cells from PCV2-infected control × 100. Data were presented as means ± SD from three independent experiments. *P < 0.05, **P < 0.01 vs PCV2-infected control

To determine the kinetics of NO production, the supernatants of the samples from 24-well cell culture plates was collected at different time points after drug treatment, and the NO levels were assayed by the Griess reaction. The NO production was significantly higher in GSNO-treated groups, with or without PVC2 infection, than the control group (*P* < 0.01; Figs. 2 and 3b). The NO levels tended to increase in the GSNO-treated groups at 0–72 h after drug treatment, reaching a plateau at around 72 h (Fig. 2). However, the NO generated from GSNO was effectively and dose-dependently scavenged by the NOscavenger Hb (Fig. 3b).

The inhibitory effect of the NO-generating compound GSNO on PCV2 replication was shown in Fig. 3. The PCV2-positive cells detected by IFA indicated that treatment with GSNO reduced the progression of PCV2 infection (Fig. 3a). The percentage of PCV2-infected cells decreased to 69.5% after treatment with 125 μM GSNO relative to the infected control (*P* < 0.05; Fig. 3c), whereas the percentage of PCV2-infected cells in the groups incubated with 125 μM GSNO plus 5, 10, or 15 μM Hb was 73.3%, 85.5% or 89.7% respectively (Fig. 3c). The viral titers and viral DNA copy numbers in the PCV2-infected groups decreased significantly after GSNO treatment relative to those in the PCV2-infected control (*P* < 0.01 or *P* < 0.05; Fig. 3d, e). However, the reductions in the viral titers and viral DNA copy numbers induced by GSNO were dose-dependently reversed by the NO scavenger Hb (Fig. 3d, e).

### Antiviral activity of GSNO in BALB/c mice

All the mice survived PCV2 inoculation with or without GSNO treatment and none of the mice were clinically affected during the study. There were no obvious gross lesions in the tissues of the experimental animals.

**Fig. 4:**
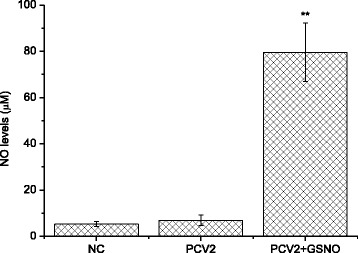
NO production in serum from the experimental mice. At 6 DPI, serum samples were obtained from six mice in each group for NO detection by Griess reaction. Data were presented as means ± SD. **P < 0.01 vs infected control

**Fig. 5:**
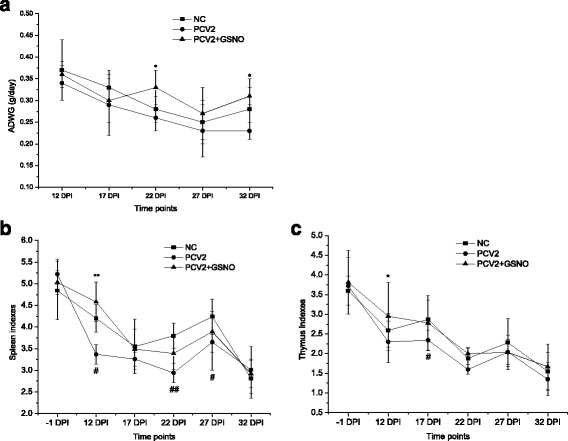
Kinetics of ADWG, SI and TI from the experimental mice. Four samples were obtained from each group at the time of necropsy. **a**  ADWG (g/day) = (final weight – initial weight) / days; **b** SI =spleen weight (g) / the weight of mice (g) × 1000. **c** TI =thymus weight (g) / the weight of mice (g) × 1000. Kinetics of ADWG, SI and TI demonstrated that, to some extent, GSNO improved growth performance and protected the immune organs (spleens and thymuses) of the mice during PCV2 infection. Data were presented as means ± SD. ^*^ *P* < 0.05, ^**^ *P* < 0.01 vs PV2-inoculated control; ^#^ *P* < 0.05, ^##^ *P* < 0.05 vs NC

GSNO treatment caused a significant increase in serum NO levels in the mice during PCV2 infection (*P* < 0.05; Fig. 4). As shown in Fig. 5a, the average daily weight gain (ADWG) tended to decrease in the PCV2-inoculated control mice at 12–32 DPI, and was lower than that in the NC mice (*P* > 0.05). However, the ADWG of the PCV2-inoculated mice treated with GSNO was significantly higher than that of PCV2-inoculated control mice (*P* < 0.05) at 22 and 32 DPI. An analysis of the kinetics of spleen index (SI) and thymus index (TI) demonstrated some GSNO-induced improvement in the mouse spleens and thymuses during PCV2 infection from −1 to 32 DPI. As shown in Fig. 5b and c, SI and TI were significantly higher in the GSNO-treated mice at 12 DPI than that in the untreated mice during PCV2 infection (*P* < 0.05 or *P* < 0.01). SI of the PCV2-inoculated control mice also decreased significantly at 12, 22 and 27 DPI compared with that of the NC mice (*P* < 0.05 or *P* < 0.01) (Fig. 5b), whereas TI decreased significantly in the PCV2-inoculated control mice at 17 DPI (*P* < 0.05; Fig. 5c). However, there were no significant differences in the SI and TI of the GSNO-treated mice and NC mice at −1―32 DPI (*P* > 0.05).

**Fig. 6:**
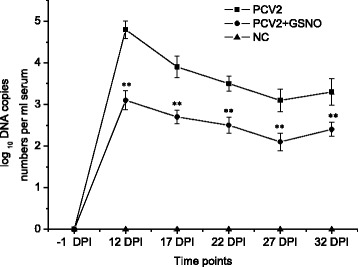
PCV2 DNA copies, detected by Taqman real-time PCR, in the sera samples from the mice with different treatments. Four sera samples were collected from each group at the time of necropsy. Data were presented as means ± SD. ** *p* < 0.01 vs PCV2-inoculated control

Throughout the experiment, all the serum samples obtained from the NC mice were negative for PCV2-specific nucleic acids when analyzed by gel-based PCR and quantitative real-time PCR assays. In the PCV2-inoculated mice, PCV2 DNA was detected by gel-based PCR in the pooled tissue samples and serum samples, and PCV2 DNA was determined in 75% (15/20) of the tissue samples and in 70% (14/20) of the serum samples from the PCV2-inoculated control mice between 12 and 32 DPI (Tables 1 and 2). However, the percentages of PCV2-positive tissue samples and serum samples from the GSNO-treated mice infected with PCV2decreased to 45% (9/20) and 40% (8/20) respectively (Tables 1 and 2). Significant differences were also noted in the PCV2-positive tissue samples (*P* = 0.012) and PCV2-positive serum samples (*P* = 0.005) from the PCV2-inoculated control mice and PCV2-inoculated mice treated with GSNO (Tables 1 and 2). As described in Fig. 6, the number of PCV2 DNA copies reached a plateau at 12 DPI, but declined to some extent between 12 and 32 DPI in the PCV2-inoculated mice treated with or without GSNO. However, at 12–32 DPI, the number of PCV2 DNA copies xwas significantly lower in the PCV2-inoculated mice treated with GSNO at each time point compared with those in the PCV2-inoculated control mice (*P* < 0.01; Fig. 6).

**Table 1:** Incidence of PCV2-positive tissue pool from the experimental mice detected by gel-based PCR analysis

Groups	12 DPI	17 DPI	22 DPI	27 DPI	32 DPI	Total
NC	0/4	0/4	0/4	0/4	0/4	0/20
PCV2	4/4	3/4	3/4	2/4	3/4	15/20
PCV2 + GSNO	2/4	2/4	2/4	1/4	2/4	9/20

The tissue pools consisted of liver, thymus, lung and spleen. Data were presented as number of PCV2 PCR positive tissue pools/total number tissue pools analyzed

**Table 2:** Incidence of PCV2 positive serum sample from the experimental mice as detected by gel-based PCR analysis

Groups	12 DPI	17 DPI	22 DPI	27 DPI	32 DPI	Total
NC	0/4	0/4	0/4	0/4	0/4	0/20
PCV2	3/4	3/4	3/4	2/4	3/4	14/20
PCV2 + GSNO	2/4	2/4	1/4	1/4	2/4	8/20

Data were presented as number of PCV2 PCR positive serum samples/number of mice sampled

## Discussion

NO, an important cellular messenger, is involved in complex and diverse functions in various physiological and pathological processes, displaying a broad spectrum of antimicrobial activities in vitro and in vivo [24]. In this study, we verified the antiviral activity of the NO-generating compound GSNO during PCV2 infection in PK-15 cells and BALB/c mice.

The in vitro experiment showed that treatment with GSNO induced significant declines in PCV2-infected cells, viral titers and viral DNA copy numbers compared with those of the PCV2-infected samples, whereas the effect of GSNO was dose-dependently reversed by the NO scavenger Hb. These results demonstrate that the NO donor GSNO exerts significant antiviral activity against PCV2 replication in PK-15 cells. Similarly, in previous studies, the NO donor SNAP has been shown to block the replication of severe acute respiratory syndrome coronavirus (SARS coronavirus) [16], porcine parvovirus [25] and porcine respiratory coronavirus [26]. Although the inhibition of PCV2 by NO was clearly demonstrated here, it is unclear whether the antiviral effect of the NO donor GSNO was actually attributable to other factors. To address this question, convincing evidence has been presented in this study. First, the data from a MTT assay clearly excluded the possibility that antiviral effects of the NO donor GSNO on PCV2 replication might have resulted from its toxicity to the cells. Second, GSNO generated NO in the culture supernatant of PK-15 cells and NO dose-dependently suppressed PCV2 replication. Finally, the inhibitory effect of GSNO on PCV2 replication was reversed by the NO scavenger, Hb. Overall, these results strongly suggest that NO, generated from the donor GSNO, inhibits the replication of PCV2 in PK-15 cells.

The mechanisms involved in the antiviral properties of NO have partly been clarified in previous studies, which have suggested that NO plays important roles in the regulation of a viral protease [27], the innate immunity of the host [15], and the synthesis of viral proteins and nucleic acids [12]. Depending on its concentration, NO exerts its antimicrobial effects in two ways: At low concentrations, NO acts as a signaling molecule that promotes the growth and activity of immune cells, whereas at high concentrations, NO covalently binds DNA, proteins and lipids, thereby inhibiting or killing the target pathogen [28]. The antiviral activity of NO against PCV2 was demonstrated in vitro by its reduction of the numbers of virus-infected cells, viral titers and DNA copy numbers. The decline in the number of virally infected cells is probably attributable to the blockage of viral penetration to the cytoplasm or to the suppression of transcriptional steps mediated by NO [29], whereas the reduced viral titers and viral DNA copy numbers are probably attributable to the inhibition of viral protein and nucleic acid synthesis by NO [12]. However, the binding, cell entry and transcription characteristics of PCV2 that are influenced by NO were not investigated in this study. Therefore, the mechanisms underlying the inhibition of PCV2 replication by NO remain to be evaluated in future studies.

Although the antiviral activity of GSNO was clearly demonstrated in vitro, in vivo studies are also essential to confirm the effects of GSNO on PCV2 infection under clinical conditions. Mice, including BALB/c, C57BL/6, C3H/HeJ and Kunming mice, are susceptible to PCV2, although susceptibility to PCV2 is limited among the above-mentioned mouse lines [30–32]. Therefore, the mouse is important in the epidemiology of PCV2 and can be used as an experimental model of PCV2. In this study, BALB/c mice were artificially inoculated with PCV2 and treated with or without GSNO. Our data showed that GSNO treatment released a large amount of NO, which contributed to the effective inhibition of PCV2 replication in the mice. The poor growth performance and immune organ (spleen and thymus) dysfunction induced by PCV2 infection were also improved to some extent by GSNO. Previous reports have demonstrated that dietary l-arginine supplementation induces significant increase in serum NO production and suppresses PCV2 infection in mice [33, 34], suggesting that the antiviral effects of l-arginine are predominantly mediated by NO because l-arginine is the sole substrate for NO synthesis [35]. This hypothesis was confirmed in our in vivo study, because GSNO was the exogenous NO donor and released NO in mice. Although no weight loss or wasting was observed in the PCV2-inoculated mice, as has been described previously, the ADWG of the mice infected with PCV2 decreased to some extent in the present study, which might be be attributable to the different mouse line used there. Since Kiupel et al. (2005) demonstrated that PCV2 induces apoptosis by activating caspases 8 and 3 in the spleens of infected mice [36]. Therefore, the impaired immune organs detected in the PCV2-inoculated control mice in our study might be attributable to the apoptosis induced by PCV2. Interestingly, the GSNO treatment improved growth performance and protected the immune organs (spleen and thymus) of the PCV2-inoculated mice. As we know, NO acts as a key molecule in inflammation and the immune response [37, 38], so the NO-generating compound GSNO may play a critical role in the regulation of inflammation and immunity during PCV2 infection, as well as inhibiting PCV2 replication, in mice. More research is required to confirm this hypothesis.

## Conclusions

GSNO, an exogenous NO donor, released NO into the culture supernatants of PK-15 cells and the sera of BALB/c mice. The antiviral effects of GSNO on PCV2 were also demonstrated in both PK-15 cells and BALB/c mice. As well as the significant inhibition of PCV2, GSNO also positively regulated the growth performance and immune organs of the PCV2-infected mice. Therefore, NO and its donor GSNO have potential valve as antiviral reagents during PCV2 infection. However, the mechanisms underlying the antiviral activity of GSNO remain to be investigated in our future studies. The effects of GSNO on PCV2 infection in the target animal, the pig, must also be evaluated.

## Abbreviations

ADWG: average daily weight gain
DPI: days post inoculation
GSNO: S-Nitrosoglutathione
Hb: hemoglobin
IFA: indirect fluorescence assay
MOI: multiplicity of infection
MTT: 3-(4,5-Dimethylthiazol-2-yl)-2,5-diphenyltetrazolium bromide
NC: negative control
NO: nitric oxide
PCR: polymerase chain reaction
PCV2: porcine circovirus type 2
PCVAD: porcine circovirus-associated disease
PDNS: porcine dermatitis and nephropathy syndrome
PMWS: post-weaning multisystemic wasting syndrome
PPV: porcine parvovirus
PRRSV: porcine respiratory and reproductive syndrome virus
SI: spleen index
SNAP: S-nitroso-acetylpenicillamine
SNP: sodium nitroprusside
TI: thymus index
